# Meaningful Words and Non-Words Repetitive Articulatory Rate (Oral Diadochokinesis) in Persian Speaking Children

**DOI:** 10.1007/s10936-016-9469-4

**Published:** 2016-12-26

**Authors:** Peyman Zamani, Hossein Rezai, Neda Tahmasebi Garmatani

**Affiliations:** Musculoskeletal Rehabilitation Research Center, Ahvaz Jundishapur University of Medical Sciences, Ahvaz, Iran

**Keywords:** Oral diadochokinesis, Meaningful word, Persian, Children

## Abstract

Repetitive articulatory rate or Oral Diadochokinesis (oral-DDK) shows a guideline for appraisal and diagnosis of subjects with oral-motor disorder. Traditionally, meaningless words repetition has been utilized in this task and preschool children have challenges with them. Therefore, we aimed to determine some meaningful words in order to test oral-DDK in Persian speaking preschool children. Participants were 142 normally developing children, (age range 4–6 years), who were asked to produce /motæka, golabi/ as two meaningful Persian words and /pa-ta-ka/ as non-word in conventional oral-DDK task. We compared the time taken for 10-times fast repetitions of two meaningful Persian words and the tri-syllabic nonsense word /pa-ta-ka/. Praat software was used to calculate the average time that subjects took to produce the target items. In 4–5 year old children, $\hbox {mean}\pm \hbox {SD}$ of time taken for 10-times repetitions of /pa-ta-ka, motæka, golabi/ were $7.72\pm 1.02, 6.58\pm 1.68$, and $6.65\pm 1.13$ seconds respectively, and in 5–6 year old children were $7.57\pm 0.95, 6.35\pm 1.38$, and $6.30\pm 1.01$ seconds respectively. Findings showed that the main effect of type of words on oral diadochokinesis was significant (${p}\,{<}\,0.001$). Children repeated meaningful words /motæka, golabi/ faster than the non-word /pa-ta-ka/. Sex and age factors had no effect on time taken for repetition of oral-DDK test. It is suggested that Speech Therapists can use meaningful words to facilitate oral-DDK test for children.

## Introduction

Speech as a complex motor function involves the coordinated processes of respiratory, phonatory and articulatory systems of the human body. Changes in speech production could be an early symptom for the existence of a motor speech disorder. Hence, using valid clinical speech assessment tools could be a great help in early diagnosis of these disorders. Although, speech phonemes including consonants and vowels (for example; mono-syllables /pa/, /ta/ and /ka/, bi-syllables /pa-ta/, /ta-ka/, or tri-syllables /pa-ta-ka/) have been used in oral-DDK test, this test is considered as a non-verbal oral task to assess the speech motor skills of children with speech disorders (Duffy 2012). This task can reveal the motor abilities of speech articulators as well as its movement limitations (such as rate and range of movement). Clearly, oral-DDK is either rated by counting the number of phonetic strings repetitions articulated in 5 or 10 sec (as ‘count-by-time’ method) or by computing the time taken for producing 10-times or more phonetic strings repetitions (as ‘time-by-count’ method) (Fletcher 1972; Gadesmann and Miller 2008). Both of these methods yield to the same result. Clinicians use this task to examine the rate and coordination between articulators such as lips, tongue and velum (Ziegler 2002). Researchers and clinicians necessarily need the reference normal values of oral-DDK rate to compare the performance of the clients in this task. Although, norm values of oral-DDK rate for repeating meaningless syllable strings are available in most languages, it can be altered by different factors like age, language context, culture, and gender. The oral-DDK values for repeating nonsense string /pa-ta-ka/ in some languages (e.g., Portuguese, English, Persian, Greek, and Hebrew) are summarized in Table 1 (Icht and Ben-David 2014; Padovani et al. 2009; Robb et al. 1985; Seifpanahi et al. 2008).

**Table 1:** Mean and standard deviation of DDK rate for repeating the meaningless three syllable sting /pataka/ in 5 different languages

Study	Language	Sample size	Mean oral DDK rate (s/10 counts) ± SD
Robb et al. (1985)	English	8	$6.25\pm 0.21$
Padovani et al. (2009)	Portuguese	23	$6.58 \pm 0.85$
Konstantopoulos et al. (2011)	Greek	27	$6.97\pm 0.85$
Seifpanahi et al. (2008)	Persian	15	$7.12 \pm 0.52$
Icht and Ben-David (2014)	Hebrew	115	$6.40\pm 0.80$

The speech materials applied in oral-DDK are usually meaningless syllabic strings (Stackhouse 2000; Yaruss and Logan 2002). Using meaningless items can negatively influence the fast and accurate performance of speech motor functions by children. Young children have challenges and difficulties with repetition of meaningless phonetic strings of oral-DDK test, so that it can lead to children’s mental confusion (Cohen et al. 1998), and it seems that rate of children’s oral-DDK productions are affected by the meaninglessness entity of the task materials. Some researchers reported that children younger than age 5 shortened the meaningless tri-syllable token of oral-DDK (/pa-ta-ka/) and it was reported that while repeating non-meaningful items, low correlations observed between children’s oral DDK rate and their articulation rates in conversation (Canning and Rose 1974). It is important that the results of oral-DDK task must be equivalent with other child’s speech motor skills. However, children articulatory errors in oral-DDK task can lead us to this question that to what extent the oral-DDK results are influenced by difficulty of the task items (Juste et al. 2012).

To answer this question, researchers revealed that young children produce more articulation errors in repeating non-word /pa-ta-ka/ than real words like /patty-cake/ or /butter-cup/. Also, they found that children articulate meaningful words faster than non-words. This could be due to the difference in the smoothness of the transition between articulatory positions, children’s familiarity with the real words versus less familiarity with the abstract nonsense syllables, or may be associated with differences in linguistic processing for non-words (Juste et al. 2012; Yaruss and Logan 2002). We found no more obvious reason to explain this difference. Given this, the current study is aimed to compare the mean time taken for 10-times repetitions of meaningful words and meaningless tri-syllable string /pa-ta-ka/ in 4–6 year old Persian speaking children. The meaningful words had comparable phonetic structure with the phonetic segment /pa-ta-ka/. We selected our participants from children between 4–6 years of age for two reasons. First, this age group has challenge to repeat the meaningless syllabic sequence of oral-DDK task. Second, having suitable oral-DDK skills to some extent indicate normal motor speech development in this age group of children, so that assessing oral-DDK abilities may yield an index of difference for differentiating children with and without motor speech disorders (Wit et al. 1993).

## Methodology

Study participants were 142, 4–6 year old children (77 girls and 65 boys), who were randomly selected from 15 kindergartens in the city of Ahvaz (south-west of Iran). Formal and informal testing were performed to clarify that none of the participated children had any speech, language, or hearing concerns, and there were no known or suspected neurological, motoric, emotional, or social problems in any of the participants. All the participants were monolingual Persian speaking children and had normal intelligence.

Selection of the meaningful words for oral-DDK test was performed in two phases. In phase one, five tri-syllable meaningful words including *golabi* (it means of /pear/ in English), *pulæki* (an Iranian candy), *pelikan* (pelican bird), *melika* (a name fit for girls in Iran), and *motæka* (pillow) were selected based on phonetic structure of the /pa-ta-ka/. With the exception of /golabi/, all the selected meaningful words have phonotactically bilabial, lingual, and velar consonants, respectively. Distinctive features of words were quantified by asking 10 volunteers with a Master of Science or PhD degree in speech-language pathology to complete a questionnaire that rates on three indicators of cultural, semantic, and phonetic context of the 5 proposed words for Persian speaking children using a 5-point scale (1 =  no desired, 2 = low desired, 3 = relatively desired, 4 = desired, 5 = highly desired). It means that, if the score of semantic fitness of a word was 5, it had highest semantic fitness. Concerning the validity of the five meaningful words, an inter-raters agreement percentage was computed for each of the 5 words and it revealed that the two words /golabi/ and /motæka/ (with an average agreement score of 87%) had correlation coefficient. In phase two, degree of children’s semantic familiarity and their fluency of articulation of two words were assessed randomly with 15 children. Each of word was three times scored at the level of 15-min intervals. Computed agreement percentage for each of the words was above than 92%. So, two meaningful words /golabi, motæka/ were used for oral-DDK test in this study.

Separately, oral-DDK rate was measured by calculating the time taken for producing ten repetitions of the tri-syllabic nonsense string /pa-ta-ka/ and two meaningful words /golabi, motæka/ as quickly as possible following an examiner’s model (time-by-count method) (Fletcher 1972). Note that only the tri-syllabic token were selected for this investigation because prior research has indicated strong correlations in oral-DDK rate between tri-, bi-, and monosyllabic tokens (Wolk et al. 1993). For each target, oral-DDK test was performed three times and the most fluent and accurate performance of the child was considered for his/her oral-DDK.

Before data collection, the examiner explained the study protocol to children’s caregivers, established an age-appropriate and child-friendly communication with participants, and provided several examples of the oral-DDK task for each child prior to data collection. The examiner used a variety of motivating techniques such as toy rewards to encourage children’s participation. All of the children received the same set of instructions for doing the oral-DDK task. The children were presented first by real words, and then, by the non-word /pa-ta-ka/. Using the script “please repeat the name of the object shown in this picture as quickly and accurately as possible, like me”, the children were prompted to repeatedly utter each of the two meaningful words. Indeed, they were shown the picture of the pear and pillow before repeating the words /golabi/ and /motæka/, respectively. Since /pa-ta-ka/ is a meaningless phonetic structure, we could not provide a picture to elicit children’s production of this non-word. Instead, examiner was saying: “please repeat /pa-ta-ka/ as fast and accurate as you can” and children were asked to repeat the the trisyllable /pa-ta-ka/. Certainly, the examiner carefully observed oral-DDK productions throughout testing so resting breaks could be provided if it became apparent that a child was beginning to fatigue. In data collection, time-by-count oral-DDK rates were audio-recorded for each participant, using a Sunny JXD/D61 digital recorder made in China placed on a table, 15 cm from the participant’s mouth. The whole session (including informed consent and debriefing) lasted approximately 15 min. Calculation of the time (per second) and 10-times of non-words versus real words repetitions was performed by version 5.4.02 of Praat software released on 26 November 2014 (Boersma 2001).

**Fig. 1:**
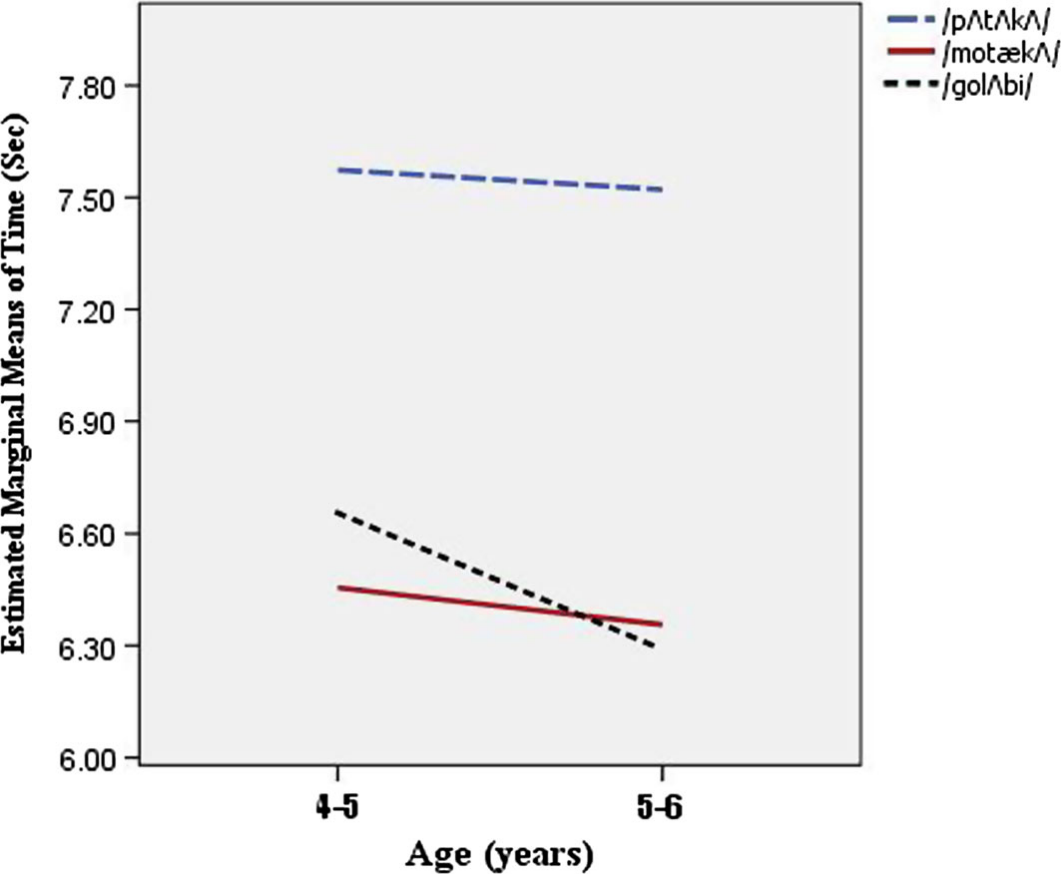
An estimation of mean time taken for 10 repetitions of the trisyllable DDK phonetic sequences in two age groups of children

## Results

142 under 6-year-old children were examined in this study. The average age of them was 4.88 years (ranging from 4–6 years of age). The mean and standard deviation (SD) of time taken for 10-times repetitions used by the male and female subjects categorized by age and sex for each tri-syllable meaningful vs. meaningless words task are presented in Fig. 1 and Table 2. As noted above, the scores are represented in second.

**Table 2:** Mean and standard deviation of time (measured in seconds) taken for 10 repetitions of trisyllable meaningful/meaningless words in 4–6 year old children categorized by age and sex

Age groups	Results of variables				
	Sex	Number	pataka	golabi	motæka
4–5 years	Girl	47	$0.87\pm 7.91$	$1.19\pm 6.76$	$1.70\pm 6.30$
	Boy	32	$1.05\pm 7.73$	$0.99\pm 6.84$	$1.58\pm 6.91$
	*p* value*		0.332	0.302	0.061
5–6 years	Girl	30	$1.02\pm 7.63$	$0.89\pm 6.53$	$1.36\pm 6.16$
	Boy	33	$1.39\pm 7.48$	$1.11\pm 6.59$	$1.42\pm 6.40$
	*p* value*		0.987	0.226	0.413

**Table 3:** Two–two comparison of the verbal DDK of trisyllable words/non-words in children ages 4–6 years

Types of words	Mean differences	SE	*P*	95% CI for mean differences lower limit -upper limit
/pataka/-/motæka/	1.252	0.147	${<}0.001$	0.896–1.609
/pataka-golabi/	1.010	0.109	${<}0.001$	0.746–1.275
/golabi-motæka/	0.242	0.113	0.105	$-$0.033–0.517

The $\hbox {mean}\pm \hbox {SD}$ of time taken for 10-times repetitions of the oral-DDK sequences /pa-ta-ka, motæka, golabi/ were $7.72\pm 1.02, 6.58\pm 1.68$, and $6.65\pm 1.13$ s respectively. In both age groups, no significant difference was observed between genders ($p\,{>}\,0.05$).

The analysis revealed that the main effect of the type of phonetic sequences (non-word/word) on the oral-DDK time was significant ($\hbox {F}_{(2,276)} = 48.01, p=0.0001, \upeta ^{2} = 0.412$). There were no other significant main effects or interactions ($p\,{>}\,0.05$). A Bonferroni PostHoc test was used to determine the significant difference between the effects of types of words used in oral-DDK tasks on the time taken for accomplishing the tasks. Findings showed that there was significant difference between the duration of 10-times repetitions of non-word /pa-ta-ka/ and the words /golabi, motæka/ ($p\,{<}\,0.001$). There is no significant difference between the duration of 10-times repetitions of meaningful words ($p\,{=}\,0.105$) (see Table 3).

## Discussion

Oral-DDK tasks are plenty accomplished in clinical settings for the appraisal of coordination of a client’s phonation and articulation systems. These tasks show a precise scale of motor disabilities involving the motor systems necessitated for speech. They have been used by Speech Therapists in diverse conventional assessment methods, as a way to evaluate motor speech disorders. In fact, the oral-DDK test is a fast and comfort tool to perform, and does not need to any special instrument. Only, fast and correct syllables repetition is desirable during oral-DDK tasks. When the rate of oral-DDK for an individual is slower than the norm (or its time taken for repetitions is greater than the norm interval), then we might be doubtful to his/her motor speech disturbances. Unfortunately, to date researchers did not study on an accepted general index to normal performance for meaningful words of oral-DDK in children. Therefore, in the current study we decided to introduce meaningful words for this task and clarify rate differences between these real words and conventional tri-syllable string /pa-ta-ka/ in 4-to-6 year old Persian speaking children.

Following the above aim, we examined a large scale sample of 142 native-Persian speakers. The mean rate of 10-times repetitions of the non-word /pa-ta-ka/ was $7.72\pm 1.02$ in age group 4-to-5 years and $7.57\pm 0.95$ s in age group 5-to-6 years which, these values were highly similar to other study (with no significant difference) in Persian language (Seifpanahi et al. 2008) (Table 1). Using Dr. Speech software, they calculated oral-DDK for nonsense phonetic sequence and reported the mean repetitions rate of the non-word /pa-ta-ka/ were $7.12\pm 0.52$ s. This suggests that the same norms and thresholds established for Persian speaking children by Dr. Speech and Praat software for /pa-ta-ka/ can be used with Persian language.

As noted above, the norm of oral-DDK values for some languages have been revealed. In a review study by Icht and her colleague, they showed that the range time of oral-DDK for meaningless phonetic strings is 5.5–7 s. (Icht and Ben-David 2014).

Findings showed that with the increase of children’s age from 4 to 6 years, the time taken for 10-times repetitions of oral-DDK phonetic sequences was reduced. Children’s oral-DDK rates increased with chronological age is consistent with prior reports. Fletcher (1972) mentioned that children’s oral-DDK rates increased with chronological age, but, he did not discuss about the existence or absence of the significant difference between the performance of the two age groups 4–5 and 5–6 years. Certainly, speech abilities and speed of articulators change during maturation periods. As children grow up, they gradually acquire new and advanced motor capabilities (e.g., speaking) and following it, their speech will be faster and more stable in movements. Beginning of these alterations are observed at around the age of under 3 years (Icht and Ben-David 2014), and by age 15 years, oral-DDK rates are similar to adult (Kent and Forner 1980).

However, we found no significant changes in time taken for 10-times repetitions of the oral-DDK tasks between age groups. We observed a relative time taken deduction in 10-times repetitions of stimuli in older children, but these differences were not significant. Indeed, these findings are novel in 4-to-6 year-old Persian-speaking children. The studies that used different methodologies, such as different tokens (e.g., /pattycake/ or /buttercup/) and different assessment procedures demonstrated miscellaneous mean rate of 10-times repetitions of the word /pattycake/. For example, the mean rate of 10-times repetitions of the word /pattycake/ has been calculated $6.49\pm 1.60$ s (Robbins and Klee 1987), and in other study has been calculated $8.26\pm 1.90$ s (McNeil 2009; Wit et al. 1993; Yaruss and Logan 2002). It can be seen; the mean values found in current study (Persian speaking children) have difference with two above studies (English speaking children). As noted above, there is no information about Persian speaking children’s meaningful word oral-DDK abilities. Due to non-existence of studies on mean time taken for repetition of the Persian meaningful words, we could not compare our findings with the similar studies in Persian speaking children. Our findings showed that regardless other variables, the main effect of meaningfulness of phonetic string on time taken of oral-DDK tasks. It means that, 4-to-6 year old children can produce the meaningful words /motæka/ and /golabi/ in significantly shorter periods of time than /pa-ta-ka/. Other studies showed that young children produce /pattycake/ and /buttercup/ (as real words) faster than /pa-ta-ka/ (as non-word) (McNeil 2009; Wit et al. 1993; Yaruss and Logan 2002), a finding that is consistent with our study.

Although, there are phonetic contexts differences in our target strings, the exact causes for children’s slowness with /pa-ta-ka/ than meaningful words is not completely apparent. One possibility of the difference between real words (such as /motæka/, and /golabi/) and non-word words such as /pa-ta-ka/ may be depended on special linguistic processing for non-words. Up to now, it is not known whether non-words would be more quick than real words because their word-finding from memory is not necessary (non-words have no need to semantic processing in repetition tasks) or whether meaningful words would be faster because of stored motor planning in memory that would facilitate articulation of oral-DDK tasks (Stackhouse 2000). On the other hand, the children’s quality and quantity of familiarities with the meaningful words (such as /motæka/, and /golabi/) and to be their less familiarity with the imaginary and meaningless phonetic strings /pa-ta-ka/ might be another cause to exist of these differences. If the familiarity of the words has effect on children’s motor functions with the oral-DDK tasks, then it must be to determine other real words which have more familiarity and more convenient to children and to their articulation (Yaruss and Logan 2002). So, the clinicians can use more familiar and easier words to assess speech motor skills by oral-DDK tasks. To this aim, researchers must evaluate and examine the rate, accuracy, fluency, familiarity and real words repetition to find appropriate phonetic strings for oral-DDK test (Andrade et al. 2010; Juste et al. 2012). We believe that our new finding could persuade Speech Therapists to use real words (such as /motæka/, and /golabi/ in assessment of oral-DDK as a diagnostic guideline of motor speech disorders, and as a scale for progressing within therapy, in 4-to-6 year old Persian speaking children.

## Conclusion

It seems that speech and language pathologists can use the two meaningful words /motæka/ and /golabi/ in oral-DDK rate measurement for 4-to-6 year old Persian speaking children. These children repeated the two meaningful words faster than the non-word /pa-ta-ka/. Also, sex and age did not significantly affect the performance of the participants in oral-DDK task.

## References

[CR1] Andrade CR, Queiroz DP, Sassi FC (2010). Electromyography and diadochokinesia-a study with fluent and stuttering children. Pró-Fono Revista de Atualização Cientifica.

[CR2] Boersma P (2001). Praat, a system for doing phonetics by computer. Glot International.

[CR3] Canning BA, Rose MF (1974). Clinical measurements of the speed of tongue and lip movements in British children with normal speech. British Journal of Disorders of Communication.

[CR4] Cohen W, Waters D, Hewlett N (1998). DDK rates in the paediatric clinic: A methodological minefield. International Journal of Language & Communication Disorders.

[CR5] Duffy, J. R. (2012). *Motor speech disorders: Substrates, differential diagnosis, and management*. Elsevier Mosby.

[CR6] Fletcher SG (1972). Time-by-count measurement of diadochokinetic syllable rate. Journal of Speech, Language, and Hearing Research.

[CR7] Gadesmann M, Miller N (2008). Reliability of speechdiadochokinetic test measurement. International Journal of Language & Communication Disorders.

[CR8] Icht M, Ben-David BM (2014). Oral-diadochokinesis rates across languages: English and Hebrew norms. Journal of Communication Disorders.

[CR9] Juste FS, Rondon S, Sassi FC, Ritto AP, Colalto CA, de Andrade CRF (2012). Acoustic analyses of diadochokinesis in fluent and stuttering children. Clinics.

[CR10] Kent RD, Forner LL (1980). Speech segment duration in sentence recitations by children and adults. Journal of Phonetics.

[CR11] Konstantopoulos, K., Charalambous, M., & Verhoeven, J. (2011). Sequential motion rates in the dysarthria of multiple sclerosis: A temporal analysis. In *The 17th international congress of phonetic sciences (ICPhS XVII)*.

[CR12] McNeil MR (2009). Clinical management of sensorimotor speech disorders.

[CR13] Padovani M, Gielow I, Behlau M (2009). Phonarticulatory diadochokinesis in young and elderly individuals. Arquivos de Neuro-Psiquiatria.

[CR14] Robb MP, Hughes MC, Frese DJ (1985). Oral diadochokinesis in hearing-impaired adolescents. Journal of Communication Disorders.

[CR15] Robbins J, Klee T (1987). Clinical assessment of oropharyngeal motor development in young children. Journal of Speech and Hearing Disorders.

[CR16] Seifpanahi S, Dadkhah A, Dehqan A, Bakhtiar M, Salmalian T (2008). Motor control of speaking rate and oral diadochokinesis in hearing-impaired Farsi speakers. Logopedics Phoniatrics Vocology.

[CR17] Stackhouse PWJ (2000). Rate, accuracy and consistency: Diadochokinetic performance of young, normally developing children. Clinical Linguistics & Phonetics.

[CR18] Wit J, Maassen B, Gabreels FJ, Thoonen G (1993). Maximum performance tests in children with developmental spastic dysarthria. Journal of Speech, Language, and Hearing Research.

[CR19] Wolk L, Edwards ML, Conture EG (1993). Coexistence of stuttering and disordered phonology in young children. Journal of Speech, Language, and Hearing Research.

[CR20] Yaruss, J. S., & Logan, K. J. (2002). Evaluating rate, accuracy, and fluency of young children’s diadochokinetic productions: A preliminary investigation. *Journal of Fluency Disorders, 27*(1), 65–85. quiz 85–66. 10.1016/s0094-730x(02)00112-2 12070876

[CR21] Ziegler W (2002). Task-related factors in oral motor control: Speech and oral diadochokinesis in dysarthria and apraxia of speech. Brain and Language.

